# Engineering of the Phytase YiAPPA to Improve Thermostability and Activity and Its Application Potential in Dephytinization of Food Ingredients

**DOI:** 10.4014/jmb.2403.03031

**Published:** 2024-06-30

**Authors:** Jing Zeng, Jianjun Guo, Lin Yuan

**Affiliations:** Institute of Microbiology, Jiangxi Academy of Sciences, Nanchang 330096, Jiangxi Province, P.R. China

**Keywords:** Phytase, surficial residue, site-directed mutagenesis, thermostability, molecular dynamics simulation

## Abstract

The aim of this study was to modify phytase YiAPPA via protein surficial residue mutation to obtain phytase mutants with improved thermostability and activity, enhancing its application potential in the food industry. First, homology modeling of YiAPPA was performed. By adopting the strategy of protein surficial residue mutation, the lysine (Lys) and glycine (Gly) residues on the protein surface were selected for site-directed mutagenesis to construct single-site mutants. Thermostability screening was performed to obtain mutants (K189R and K216R) with significantly elevated thermostability. The combined mutant K189R/K216R was constructed via beneficial mutation site stacking and characterized. Compared with those of YiAPPA, the half-life of K189R/K216R at 80°C was extended from 14.81 min to 23.35 min, half-inactivation temperature (*T*_50_^30^) was increased from 55.12°C to 62.44°C, and *T_m_* value was increased from 48.36°C to 53.18°C. Meanwhile, the specific activity of K189R/K216R at 37°C and pH 4.5 increased from 3960.81 to 4469.13 U/mg. Molecular structure modeling analysis and molecular dynamics simulation showed that new hydrogen bonds were introduced into K189R/K216R, improving the stability of certain structural units of the phytase and its thermostability. The enhanced activity was primarily attributed to reduced enzyme-substrate binding energy and shorter nucleophilic attack distance between the catalytic residue His28 and the phytate substrate. Additionally, the K189R/K216R mutant increased the hydrolysis efficiency of phytate in food ingredients by 1.73–2.36 times. This study established an effective method for the molecular modification of phytase thermostability and activity, providing the food industry with an efficient phytase for hydrolyzing phytate in food ingredients.

## Introduction

Phytate is the primary form of phosphorus in grasses and legumes [1, 2]. It can form stable and difficult-to-degrade complexes with proteins, minerals, and other substances in organisms, reducing the bioavailability of proteins and mineral elements [1, 2]. For this reason, phytate is considered an anti-nutritional factor. Phytase, also known as myo-inositol hexakisphosphate phosphohydrolase, can degrade phytate and generate inorganic phosphorus and phosphoinositides, eliminating the anti-nutritional effects of phytate [3, 4]. Owing to the lack of phytase in the human digestive system, phytate cannot be metabolized, which limits the absorption of nutrients in the intestine and reduces the nutritional value of food. Phytase can reduce the phytate level in food more effectively than cooking, boiling, fermentation, soaking, and other food processing methods. Adding phytase to plant-based food ingredients can degrade phytate and release inorganic phosphorus, which can improve the absorption and utilization of phosphorus in food, relieving the anti-nutritional effect of phytate [5-8]. Thus, phytase can be used as a new type of food additive in the food industry. However, numerous processing operations in the food industry necessitate execution under high temperatures. The limited thermostability of natural phytase poses a critical constraint in its practical utilization in food processing [9, 10]. Hence, the pursuit and advancement of improved thermostability of phytases remain a focal point of research among scholars worldwide.

Currently, protein engineering strategies used to enhance the thermostability of phytase include directed evolution (irrational design), semi-rational design, and rational design [11-14]. Rational design in the context of enzyme engineering involves directed modifications of enzymes based on the structure-activity relationship between molecular structure and function. Common methods employed in rational design include ion binding design, disulfide bond design, glycosylation site design, homology modeling, and molecular dynamics simulation [15-18]. For example, Sanchez-Romero *et al*. [19] introduced three pairs of disulfide bonds into the phytase CbAppA derived from *Citrobacter braakii*; this increased the denaturation temperature of CbAppA by 12.1°C. Li *et al*. utilized the molecular structure of a phytase derived from *Escherichia coli* to analyze the B-factor (temperature factor) of amino acid residues in the phytase using the software B-FITTER. Based on this analysis, they identified 13 amino acid residues as mutation sites. A mutant library was constructed, and the mutant P56214 with improved thermostability was obtained. After incubation at 90°C for 5 min, the residual activity of the phytase increased from 20% to 75% [20]. The strategy of protein surficial residue mutation is also a part of rational design methodology. By comparing the molecular structures of thermophilic and mesophilic proteins, it was found that thermophilic proteins have a characteristic distribution of arginine (Arg) replacing lysine (Lys) and alanine (Ala) replacing glycine (Gly) on their surfaces, which contributes to their thermostability.

The strategy of protein surficial residue mutation was devised based on the above findings. Firstly, the GetArea software (http://curie.utmb.edu/getarea.html) was utilized to predict the localization of Gly and Lys residues on the protein surface. Next, each Gly residue was systematically substituted with Ala, and each Lys residue was replaced with Arg, leading to the construction of a mutant library. Directed mutagenesis was accomplished by carefully screening the resulting library to obtain mutants with specific modifications. Pang *et al*. implemented protein surficial residue mutation by substituting Lys (K) at 419 with Arg (R) in a type II pullulanase (PulA) derived from *Anoxybacillus* sp. WB42. This mutation resulted in a variant, K419R, with enhanced thermostability [21]. Currently, there is no relevant research on improving phytase thermostability through amino acid mutations on protein surfaces.

The phytase YiAPPA (EC 3.1.3.2) derived from *Yersinia intermedia* is currently known as the most active phytase, with an enzymatic activity of up to 3960 U/mg (37°C, pH 4.5) [22, 23]. YiAPPA has outstanding properties such as high stability and enzymatic activity under acidic conditions and strong resistance to protease. Therefore, it has immense potential for applications in the food industry. However, the thermostability of YiAPPA (with a half-life of only approximately 30 min at 55°C) limits its application in the food industry. The aim of this study was to improve the thermostability of YiAPPA. Protein surficial residue mutation was employed to directly modify YiAPPA to obtain mutants with improved thermostability. Molecular dynamics simulation and molecular structural modeling analysis were adopted to explore the molecular mechanism for enhanced thermostability and provide a basis for the thermostability modification of phytase and other enzymes, offering effective phytases for phytate degradation in the food industry.

## Materials and Methods

### Strains, Plasmid, and Culture Growth

*Escherichia coli* JM109, *Bacillus subtilis* WB600, and recombinant plasmid pSTOP1622-*yiappah* were derived from our laboratory. Bacteria were grown at 37°C in Luria–Bertani (LB) medium containing kanamycin (30 μg/ml), as needed.

### Bioinformatic Analysis

The protein structure of the phytase YiAPPA was predicted using homology modeling by SWISS-MODEL (http://swissmodel.expasy.org) [24], with the molecular structure of the phytase YkAPPA from *Yersinia kristensenii* (PDB ID: 4ARV) as the template [25, 26]. The protein structure was then visualized using the 3D-imaging software PyMOL v0.99. The protein molecular structure model of YiAPPA was submitted to the online protein surficial residue distribution prediction software GetArea for predicting the locations of Gly and Lys residues on the surface of YiAPPA, thereby determining the potential amino acid mutation sites in YiAPPA.

### Construction and Identification of Site-Specific Mutants

The primers were designed based on the base sequence of YiAPPA and the mutated amino acid sites by referring to the instructions of the QuickMutation kit, as shown in Table S1. Taking the construction of the site-directed mutant G16A as an example, the primers G16A-F and G16A-R were used to amplify linear fragments containing the vector sequence and the mutated gene sequence from the template recombinant plasmid pSTOP1622-*yiappah* through polymerase chain reaction (PCR) amplification. The PCR amplification conditions were as follows: 95°C for 5 min; 35 cycles at 95°C for 30 s, 58°C for 30 s, and 68°C for 3 min, followed by a final extension at 68°C for 10 min. After treating the PCR amplicons with the restriction enzyme DpnI, they were directly transformed into *E. coli* JM109 cells, which were then plated on LB agar plates (kanamycin) for selection. Transformants were picked from the kanamycin-resistant agar plates, and the recombinant plasmids were extracted and sent to Shanghai Sangon Biotech Co., Ltd. for sequencing. The obtained sequences were then aligned with the reference gene for confirmation. Other site-directed mutants were constructed following the method used for mutant G16A construction.

### Induction, Expression, and Purification of Recombinant Phytase

The recombinant plasmid was transferred into competent *B. subtilis* WB600 cells to obtain recombinant *B. subtilis*. The preparation and transformation of competent *B. subtilis* WB600 cells were performed using an improved Spizizen method [27]. The induction, expression, and purification of recombinant phytase were carried out according to a previous report [23]. Sodium dodecyl sulfate-polyacrylamide gel electrophoresis (SDS-PAGE)[28] was employed to assess the purity of the recombinant phytase, based on the band pattern of protein samples displayed on the SDS-PAGE gel. The concentration of the recombinant phytase was evaluated using the Bradford method [29].

### Determination of the Recombinant Phytase Activity

A 250-μl aliquot of the phytase solution (0.005 mg/ml) was diluted and thoroughly mixed with 750 μl of 0.25 mol/l sodium acetate buffer solution (pH 4.5). Thereafter, 2 ml of 1.5 mmol/l sodium phytate solution (0.25 mol/l sodium acetate buffer, pH 4.5) was added to the experimental group, and 2 ml of the end-point mixture (ammonium molybdate/ammonium vanadate/nitric acid) was added to the control group. The mixtures were thoroughly mixed and incubated at 37°C for 30 min. Subsequently, 2 ml of the end-point mixture was immediately added to the experimental group, and 2 ml of 1.5 mmol/l sodium phytate solution was added to the control group. All samples were thoroughly mixed and tested for their absorbance at 415 nm. The unit of phytase activity (U) was defined as the amount of phytase required to release 1 μmol/l of inorganic phosphorus from the 1.5 mmol/l sodium phytate solution per minute at pH 4.5 and 37°C.

### Determination of the Enzymatic Properties of the Recombinant Phytase

The optimal reaction temperature, stability at 80°C, optimal reaction pH, stability at different pH levels, and protease resistance of the recombinant phytase were determined as previously reported [23].

The reaction system mentioned above was subjected to a 30-min reaction at temperatures ranging from 30°C to 90°C. The enzymatic activity of the samples was measured at various temperatures. The highest enzymatic activity was defined as 100%, and relative enzymatic activity was calculated for each temperature. A graph was plotted with relative enzymatic activity against temperature to determine the optimal reaction temperature. After incubating the enzyme solution at 80°C for 0–40 min, the enzymatic activity of the samples was determined according to the reaction system described above. The residual activity of the other samples was calculated using the enzymatic activity of the untreated sample as 100% and was plotted against temperature to evaluate the stability of the enzyme at 80°C.

The enzymatic activity at different pH levels (1.0–8.0) was determined. The relative enzymatic activity was calculated using the highest value as 100% enzymatic activity and was plotted against temperature to determine the optimal reaction pH. The enzyme solution was diluted using buffers of various pH levels (1.0–12.0), followed by a 2-h treatment at 37°C. Subsequently, the samples were diluted using the buffer with the optimal pH. The enzymatic activity of each sample was then measured based on the reaction system described above. The residual activity of other samples was calculated by using the enzymatic activity of the untreated sample as 100%, and the residual activity-temperature graph was plotted to evaluate the stability of enzymes under different pH conditions.

Solutions of 0.1 mg/ml of pepsin (0.25 mol/l Gly-HCl buffer, pH 2.0) and trypsin (0.25 mol/l Tris-HCl buffer, pH 7.0) were separately prepared. According to a proteinase-to-phytase mass ratio of 1:10, pepsin and trypsin were added to the phytase solution. The mixtures were then incubated at 37°C for 2 h, and a proteinase inhibitor was added to stop the reaction. Subsequently, the samples treated with proteinase were diluted 100-fold using the optimal pH buffer (0.25 mol/l sodium acetate buffer, pH 4.5). The enzymatic activity of the phytase before and after treatment with proteinase was determined using the reaction system described above.

### Determination of Thermodynamic Parameters of the Recombinant Phytase

The thermodynamic parameter characterization of recombinant phytase mainly includes the determination of its half-life (*t*_1/2_), *T*_50_^30^, and melting temperature (*T*_m_). The enzyme solution (0.005 mg/ml) was incubated at 80°C, and the enzymatic activity was measured at different time points. The residual activity of the other samples was calculated with the enzymatic activity of the untreated sample as 100%. Linear regression was performed by plotting the logarithm of residual activity on the y-axis against time (t) on the x-axis to calculate the deactivation rate constant (*k*_d_) of the recombinant phytase. The *t*_1/2_ of the recombinant phytase was calculated using the first-order deactivation Eq. (1) [30, 31], where A_0_ represents the initial enzymatic activity and A_t_ represents the enzymatic activity at different time points.

$${\text{A}}_{\text{t}}{\text{=A}}_{\text{0}}{\text{e}}^{{\text{-k}}_{\text{d}}\text{×t}}$$ (1)

After incubating the enzyme solution (0.005 mg/ml) at 45°C, 50°C, 55°C, 60°C, 65°C, and 70°C for 30 min, the enzymatic activity of the samples was measured. Thereafter, the residual activity of the samples incubated at various temperatures was calculated with the enzymatic activity of the untreated sample as 100%. The temperature at which the residual activity was 50% was regarded as the *T*_50_^30^.

The *T*_m_ of the recombinant phytase was measured using Nano-DSC. First, the sample was degassed and loaded. After the heat flow and pressure were stabilized, the sample was scanned by increasing the temperature from 30°C to 80°C at a rate of 1°C/min. The *T*_m_ was then obtained by fitting the test structure using the Nano Analysis software.

### Determination of Kinetic Constant of the Recombinant Phytase

Sodium phytate solutions of various concentrations (0.0625%, 0.1%, 0.125%, 0.2%, 0.25%, 0.5%, 1.0%, and 1.5%) were prepared by diluting the stock solution with 0.25 mol/L sodium acetate buffer solution (pH 4.5). Equal volumes of phytase solution were added to each sodium phytate solution, and the enzymatic activity was determined following the method described above. Based on the double reciprocal plot, a graph was constructed with the reciprocal of substrate concentration as the x-axis and the reciprocal of enzyme activity as the y-axis. The slope of the line was *K_m_*/*V*_max_ and the intercept was 1/*V*_max_. The Michaelis constant (*K_m_*), maximum reaction velocity (*V*_max_), and reaction constant (*k*_cat_) were calculated with the sodium phytate as the substrate.

### Molecular Dynamics Simulation and Molecular Docking

Molecular dynamics simulations were performed using Gromacs 2019.6 [32] at a temperature of 353 K (80°C) for a duration of 100 ns to analyze the thermodynamic fluctuations of the recombinant phytase. The simulations were conducted under constant temperature and pressure using the Amber14SB all-atom force field and the TIP3P water model. Periodic boundary conditions were applied during the simulations. The RMSD file reflecting the structure rigidity of the phytase molecule and the RMSF file reflecting the fluctuation of amino acid residues in phytase were obtained after the operation.

The recombinant phytase and phytate substrate were subjected to molecular docking using the software AutoDock 4.2.6 [33]. The binding energy and different energy contributions of the recombinant phytase and phytate substrate were calculated using the MMPBSA method [34]. Additionally, the software PyMOL v0.99 was employed to analyze the structural variations of the recombinant phytase at crucial amino acid residues.

### Dephytinization of Food Ingredients

A 50-ml aliquot of 0.25 mol/l sodium acetate buffer (pH 4.5) was added to 5 g of food ingredients (wheat, corn, and soybean flours). After thoroughly mixing the samples, 10 U of purified recombinant phytase was added to the mixture. The mixture was then incubated at 55°C and 100 rpm for 4 h. Aliquots of samples were collected at 0.5, 1, and 2 h, and tested for the content of inorganic phosphorus and phytate as previously reported [35].

### Statistical Analysis

Each of the enzymatic property tests was conducted in triplicate. Statistical analysis of experimental data was carried out using SigmaPlot 12.5, and all data are expressed as mean ± standard deviation.

## Results and Discussion

### Construction and Screening of Single-Site Mutants

This study intended to improve the thermostability of the phytase YiAPPA using protein surficial residue mutation. According to this molecular engineering strategy, the Lys and Gly residues located on the surface of YiAPPA were first analyzed. The amino acid sequence of YiAPPA was submitted to SWISS-MODEL for homology modeling using the molecular structure of the phytase YkAPPA (PDB ID: 4ARV) as the template, obtaining the molecular structure model for YiAPPA. The molecular structure model of YiAPPA was then submitted to the online prediction software GetArea. According to the working principle of GetArea, amino acid residues with a surface exposure ratio value greater than 50% were considered to be located on the surface of the protein molecule. The surficial Lys and Gly residues of YiAPPA predicted by GetArea are displayed in Table 1.

**Fig. 1:**
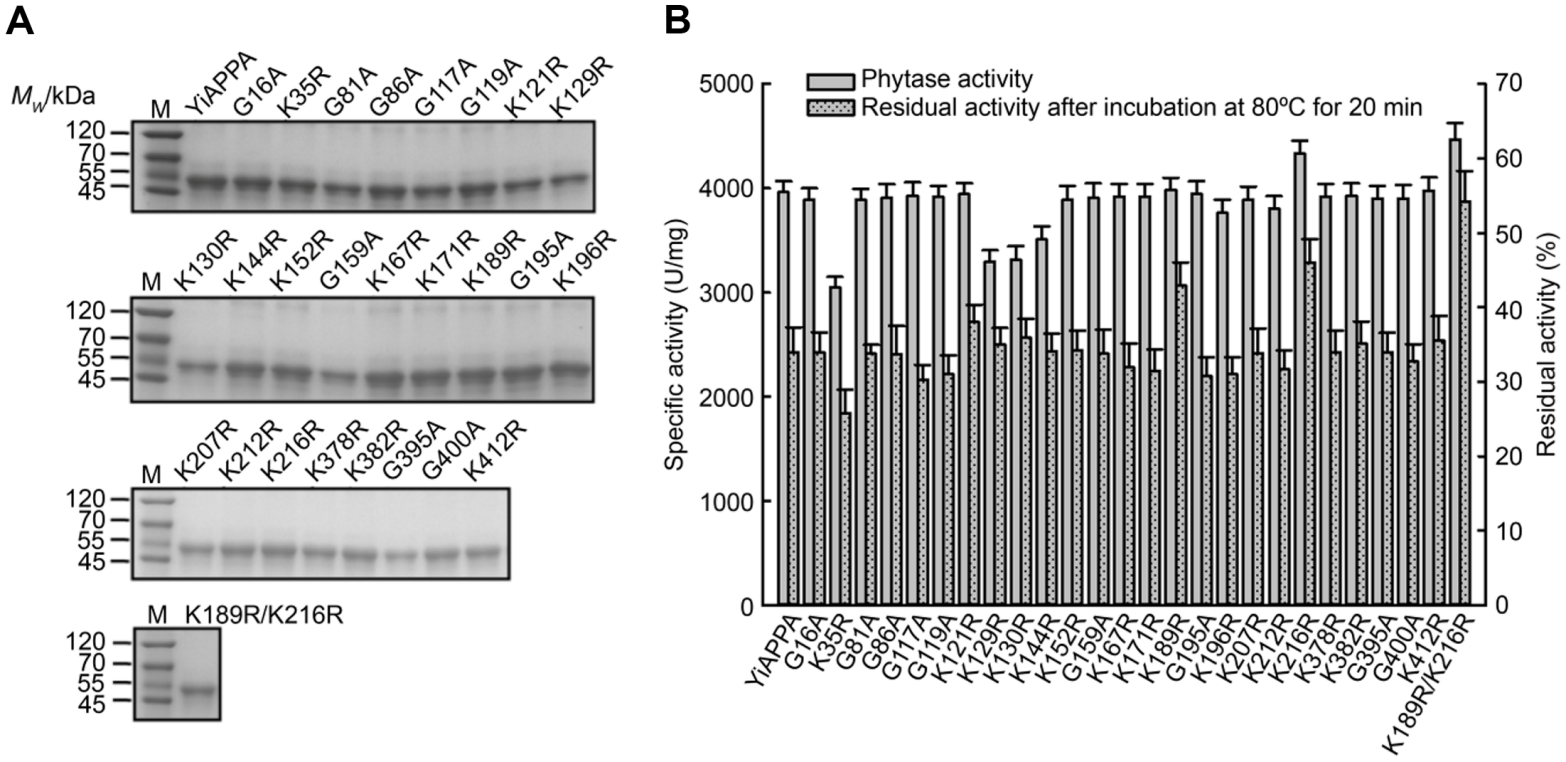
SDS-PAGE analysis and enzymatic activity and thermostability determination of the recombinant phytase YiAPPA and its single-site mutants. (**A**) M. Protein marker; YiAPPA, G16A, K35R, G81A, G86A, G117A, G119A, K121R, K129R, K130R, K144R, K152R, G159A, K167R, K171R, K189R, G195A, K196R, K207R, K212R, K216R, K378R, K382R, G395A, G400A, K412R, and K189R/K216R are purified recombinant phytases.

Based on the prediction results in Table 1, YiAPPA mutants by replacing surface Lys with Arg (K35R, K121R, K129R, K130R, K144R, K152R, K167R, K171R, K189R, K196R, K207R, K212R, K216R, K378R, K382R, and K412R) and by replacing surface Gly with Ala (G16A, G81A, G86A, G117A, G119A, G159A, G195A, G395A, and G400A) were constructed. The heterologous recombination and expression of YiAPPA and its single-site mutants were carried out in *B. subtilis* WB600. Subsequently, the expressed recombinant phytase was isolated and purified. The purity of the recombinant phytases was determined using SDS-PAGE analysis. Furthermore, the specific activity and residual activity after incubating the samples at 80°C for 20 min were evaluated. The results presented in Fig. 1A show the successful recombination and expression of YiAPPA and its mutants; a single-band product was obtained through purification. SDS-PAGE analysis showed that the molecular weight of YiAPPA and its mutant was approximately 48 kDa, the same as that of wild-type YiAPPA [22].

Fig. 1B shows that the specific activity of recombinant YiAPPA was 3960.81 U/mg. The specific activity of the K216R mutant was 4352.96 U/mg, which was 1.10 times higher than that of recombinant YiAPPA. The specific activity of the K35R, K129R, K130R, and K144R mutants was significantly lower than that of YiAPPA. The specific activity of the other mutants was almost the same as that of recombinant YiAPPA. The residual activity of the mutants K189R and K216R after 20 min at 80°C was 43.00% and 46.00%, respectively, significantly higher than that of recombinant YiAPPA (33.99%). The above results demonstrated that the molecular engineering of the phytase YiAPPA through protein surficial residue mutation generated two mutants: K189R, with significantly improved thermostability, and K216R, with enhanced enzymatic activity and thermostability.

### Construction and Enzymatic Property Analysis of K189R/K216R

Construction of K189R/K216R and the Preliminary Characterization of Its Enzymatic Properties

Mutation stacking is a method to improve enzymatic properties by combining beneficial mutations [36, 37]. Based on the previous study results, in this study, two mutants with significantly enhanced thermostability were stacked, constructing a combined mutant K189R/K216R. The combined mutant K189R/K216R was introduced into *B. subtilis* WB600 for heterologous recombination and expression. The recombinant phytase was then expressed, purified, and subjected to preliminary characterization for its enzymatic properties. As shown in Fig. 1A, the combined mutant K189R/K216R was successfully expressed by *B. subtilis* WB600, and its molecular weight determined using SDS-PAGE was consistent with that of recombinant YiAPPA. Fig. 1B and Table 2 show that the specific activity (4469.13 U/mg) and thermostability (54.23%, after being incubated at 80°C for 20 min) of the K189R/K216R mutant were further improved, which were 1.13 times that of recombinant YiAPPA and 0.60 times higher than that of recombinant YiAPPA, respectively. Wang *et al*. introduced three N-glycosylation sites into the phytase YiAPPA, and the specific activity of the resulting mutant M14 was basically unchanged but the thermostability was considerably improved. The residual activity of the mutant M14 reached 76% after 20 min at 80°C [15]. The thermostability of K189R/K216R obtained in this study was not as good as that of the mutant M14, but both its specific activity and thermostability were improved. In this study, the optimal reaction temperature, thermodynamic parameters, optimal reaction pH and pH stability, kinetic parameters, and protease resistance of K189R/K216R were further determined.

### Determination of the Optimal Reaction Temperature and Thermodynamic Parameters for K189R/K216R

**Fig. 2:**
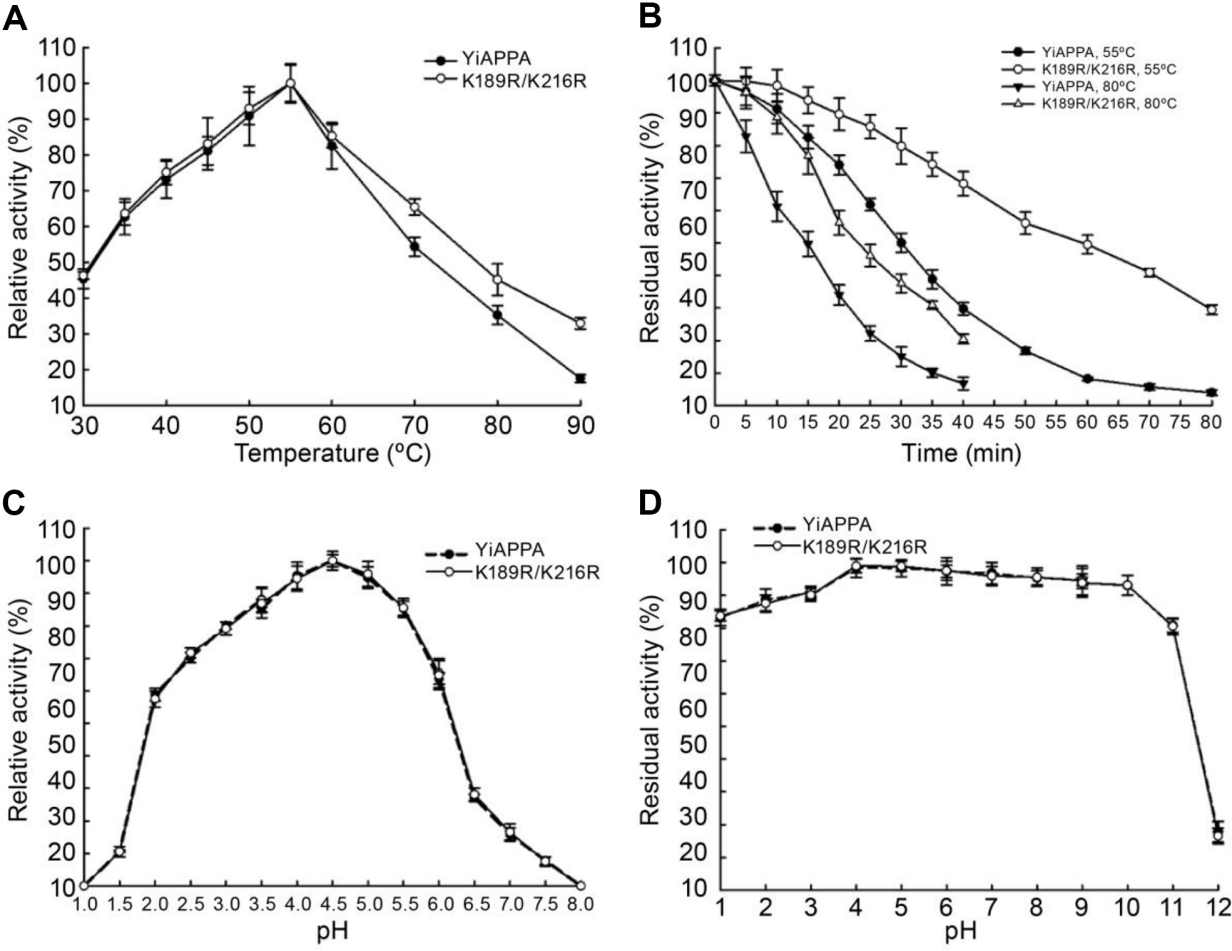
Effect of temperature and pH on the activity and stability of the recombinant phytase. (**A**) The effect of temperature on the activity of the recombinant phytase. (**B**) The effect of temperature on the stability of the recombinant phytase. (**C**) The effect of pH on the activity of the recombinant phytase. (**D**) The effect of pH on the stability of the recombinant phytase.

This study determined the optimal reaction temperature and thermodynamic parameters of the recombinant phytase YiAPPA and its mutant K189R/K216R, and the results are shown in Fig. 2 and Table 2. Fig. 2A shows that the optimal reaction temperature of the recombinant phytase YiAPPA and K189R/K216R mutant was 55°C. The relative activity of YiAPPA was similar to that of K189R/K216R within the range of 30°C–55°C. However, at 60°C–90°C, the relative activity of K189R/K216R was significantly higher than that of YiAPPA. The above results indicate that K189R/K216R retained higher relative activity within the higher temperature range, and this may be associated with its superior thermostability. Fig. 2B reveals that the residual activity of K189R/K216R was significantly higher than that of YiAPPA after incubation at 55°C or 80°C, indicating its significantly higher stability at 55°C and 80°C than YiAPPA.

Based on these investigations, the thermodynamic parameter characterization of the recombinant phytase was carried out by determining the *t*_1/2_, *T*_50_^30^, and *T*_m_. The results are shown in Table 2. The *t*_1/2_ of recombinant YiAPPA at 55°C was determined to be 30.09 min. Relative to the recombinant YiAPPA, the *t*_1/2_ of K189R/K216R, K189R, and K216R were increased by factors of 1.01 (60.45 min), 0.57 (47.34 min), and 0.66 (50.09 min), respectively, under the same temperature conditions. At a higher temperature of 80°C, the *t*_1/2_ for K189R/K216R was 23.35 min, which represents a 0.58-fold increase relative to YiAPPA (14.81 min). Similarly, the *t*_1/2_ of K189R (17.86 min) and K216R (18.90 min) at 80°C were increased by 0.21-fold and 0.28-fold, respectively, compared to that of recombinant YiAPPA. The *T*_50_^30^ of K189R/K216R was 62.44°C, which was 7.32°C higher than that of recombinant YiAPPA (55.12°C). The *T*_50_^30^ of K189R (57.32°C) and the *T*_50_^30^ of K216R (59.85°C) were 2.20°C and 4.73°C higher than those of recombinant YiAPPA, respectively. The *T*_m_ of K189R/K216R was 53.18°C, which was 4.82°C higher than that of recombinant YiAPPA (48.36°C). The *T*_m_ of K189R (49.93°C) and the *T*_50_^30^ of K216R (50.79°C) were 1.57°C and 2.43°C higher than those of recombinant YiAPPA, respectively. In conclusion, the thermostability of the mutant K189R/K216R was significantly improved compared with that of recombinant YiAPPA.

### Determination of the Optimal Reaction pH and pH Stability of K189R/K216R

This study determined the optimal reaction pH and the pH stability of the recombinant phytase YiAPPA and its mutant K189R/K216R, and the results are shown in Fig. 2C and 2D. The optimal reaction pH of the recombinant phytase YiAPPA and K189R/K216R mutant was 4.5, and they yielded similar relative enzyme activities at pH 1.0–8.0 (Fig. 2C). Within the pH range of 1.0–12.0, the stability of the recombinant phytase YiAPPA and K189R/K216R mutant was also similar (Fig. 2D). Within the pH range of 1.0–3.0, both exhibited over 80% residual enzyme activity. Within the pH range of 4.0–10.0, both demonstrated over 90% relative enzyme activity. However, within the pH range of 11.0–12.0, both were unstable and rapidly inactivated.

### Determination of the Kinetic Parameters of K189R/K216R

The specific activity, *K_m_*, and *k*_cat_ of the recombinant phytase using sodium phytate as the substrate are shown in Table 2. The specific activity of the K189R/K216R mutant was 0.13 times higher than that of recombinant YiAPPA. The *K_m_* value of K189R/K216R was 111.59 μM, which was 11.60% lower than that of YiAPPA (126.24 μM). The *k*_cat_ value of YiAPPA was 10375.27 s^-1^, similar to that of K189R/K216R (10363.29 s^-1^). The specific activity, *K_m_* value, and *k*_cat_ value of the K189R mutant were similar to those of YiAPPA. Additionally, the specific activity of the K216R mutant was 0.10 times higher than that of YiAPPA. The *K_m_* value of K216R was 115.01 μM, which decreased by 8.90% compared to that of YiAPPA. The *k*_cat_ value of K216R was 10381.09 s^-1^, similar to that of YiAPPA. Based on the above results, the mutation of Lys^-1^89 (K) to Arg (R) in YiAPPA did not have an observable impact on its substrate binding ability and substrate catalytic efficiency, which is consistent with the unaltered enzymatic activity observed in K189R. On the other hand, replacing Lys-216 (K) with Arginine (R) solely enhanced its substrate binding ability without affecting its substrate catalytic efficiency, resulting in an increased enzymatic activity for K216R. Furthermore, when both mutations of Lys^-1^89 (K) to Arg (R) and Lys-216 (K) to Arg (R) were combined in YiAPPA, it further augmented its substrate binding ability while maintaining unchanged substrate catalytic efficiency, thereby confirming that the mutant K189R/K216R exhibited the highest enzyme activity.

### Determination of Protease Resistance of K189R/K216R

The results of the protease resistance analysis of the K189R/K216R mutant are shown in Table 2. The recombinant phytase YiAPPA and its mutants K189R, K216R, and K189R/K216R showed comparable protease resistance. Following pepsin treatment, recombinant YiAPPA retained 84.17% residual enzymatic activity, while the mutants K189R, K216R, and K189R/K216R retained 84.36%, 84.91%, and 85.43% residual enzymatic activity, respectively. After trypsin treatment, recombinant YiAPPA retained 82.28% residual enzymatic activity, whereas the mutants K189R, K216R, and K189R/K216R retained 83.01%, 83.30%, and 83.97% residual enzymatic activity, respectively. Overall, the introduction of mutations (K189R and/or K216R) in YiAPPA did not significantly affect its resistance to proteases.

### Exploration of the Molecular Mechanism of Increased Thermostability and Activity of K189R/K216R

**Fig. 3:**
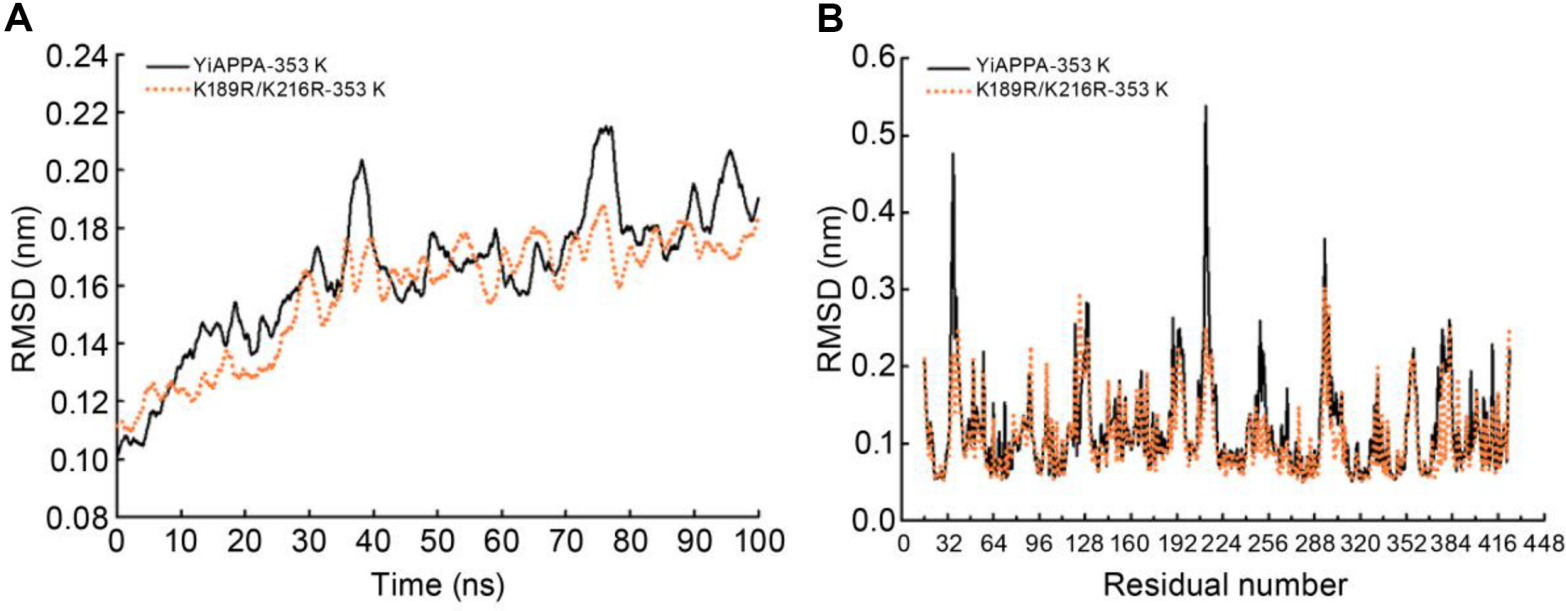
Molecular dynamics simulation results of the recombinant phytase. (**A**) RMSD value analysis of the recombinant phytase YiAPPA and K189R/K216R at 353 K (80°C). (**B**) RMSF value analysis of the recombinant phytase YiAPPA and K189R/K216R at 353 K (80°C).

**Molecular dynamics simulation of the recombinant phytase.** At 353 K, a 100-ns molecular dynamics simulation was performed on the tertiary structure of YiAPPA and K189R/K216R to analyze their thermodynamic fluctuations, and the overall structural rigidity of the enzyme molecule and the fluctuations of amino acid residues were analyzed. The results are shown in Fig. 3. Fig. 3A shows that the RMSD value of YiAPPA and K189R/K216R fluctuated less after 40 ns and reached equilibrium, with the mean RMSD value of YiAPPA being 0.18 Å and that of K189R/K216R being 0.17 Å, that is, the mean RMSD value of the mutant K189R/K216R was slightly lower than that of YiAPPA. The RMSD value of an enzyme depends on its overall structural rigidity and stability. The lower the RMSD value, the greater the structural rigidity of the enzyme molecule, showing that it is more stable. The K189R/K216R mutant has a smaller RMSD value than YiAPPA, indicating it has a more stable molecular structure.

Fig. 3B presents the RMSF analysis results of YiAPPA and K189R/K216R. At Lys^-1^89 and its nearby residues (serine (Ser)-190 to cysteine (Cys)-198, S190-C198), the RMSF value of K189R/K216R decreased compared with that of YiAPPA, showing that the surrounding amino acid residues became more stable due to the mutation of Lys^-1^89 in YiAPPA. This finding means that this single-site mutation also improved the stability of amino acid residues at nearby sites. In the region adjacent to the mutation site of Lys-216 (K216) (threonine (Thr)-209 to Cys-214, T209-C214, valine (Val)-217 to leucine (Leu)-219, V217-L219), the RMSF value of K189R/K216R was significantly lower than that of YiAPPA. However, the RMSF of the K216 site was unchanged, indicating that although Lys mutation at position 216 in YiAPPA, as an essential mutation site, did not alter its RMSF, it improved the stability of amino acid residues in the nearby region.

These findings demonstrate that K189R/K216R had a slightly lower average RMSD value than YiAPPA, and the RMSF value of its mutation and nearby sites was significantly reduced. The molecular structure of K189R/K216R was more rigid, and some of its amino acid residues were more stable, leading to the higher thermostability of K189R/K216R than that of YiAPPA.

**Fig. 4:**
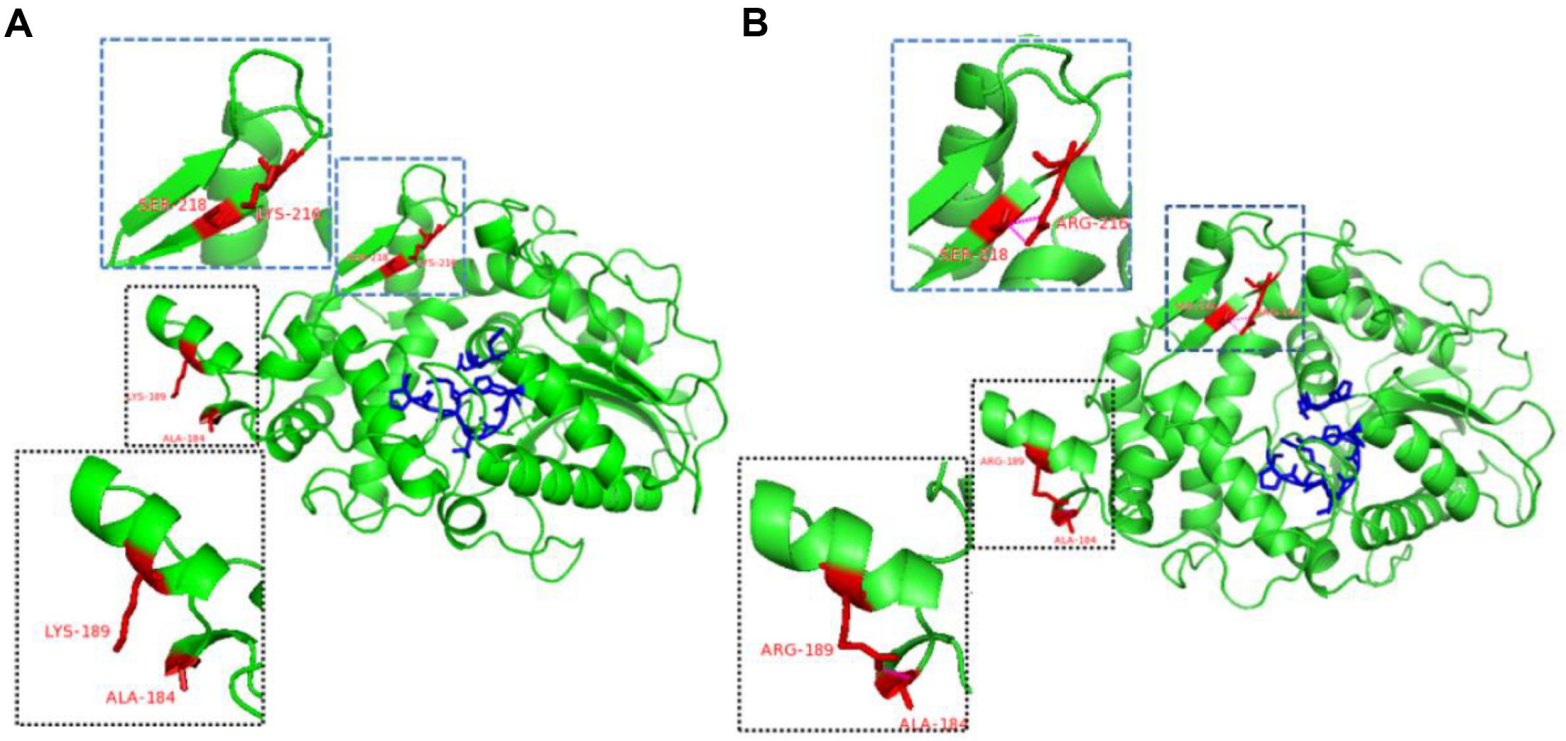
Molecular structure analysis of the recombinant phytase. (**A**) Molecular structure analysis of the recombinant phytase YiAPPA. (**B**) Molecular structure analysis of the recombinant phytase K189R/K216R. The blue parts in the figure represent the amino acid residues constituting the active site of the phytase. The red parts indicate the mutated amino acid residues (Lys^-1^89 mutated to Arg-189; Lys-216 mutated to Arg-216) and the amino acid residues forming new hydrogen bonds with them (Ala-184 forms a new hydrogen bond with Arg-189; Ser-218 forms a new hydrogen bond with Arg-216). Hydrogen bonds are outlined in the magenta dashed box.

**Molecular structure analysis of the recombinant phytase.** Using the structure of the phytase YkAPPA (PDB ID 4ARV) as the template, the structure models of YiAPPA and K189R/K216R were constructed through homologous modeling, and the interaction forces between the amino acid residues of mutation sites and their surrounding sites before and after mutation were analyzed to explore the reasons for the increased rigidity of K189R/K216R. The results are shown in Fig. 4A and 4B. Lys^-1^89 is located in the α-helix away from the catalytic pocket and has a long and flexible side chain. When Lys^-1^89 was mutated to Arg, a hydrogen bond was formed between the newly added Lys at position 189 and the Ala at position 184. Based on the results of the RMSF analysis, it can be concluded that the mutation of Lys to Arg at position 189 in YiAPPA further stabilized both itself and the Ser at position 190 to Cys at position 198. This increase in hydrogen bonding between the newly added R189 (Arg-189) and A184 (Ala-184) enhanced the conformational stability of this protein region. As shown in Fig. 4A and 4B, Lys-216 is located in the loop structure near the catalytic pocket, and its mutation to Arg introduced a hydrogen bond between Arg-216 and Ser-218. The results of molecular dynamics simulation revealed significantly reduced RMSF values for Thr-209 to Cys-214 and Val-217 to Leu-219. Specifically, this decrease indicates enhanced rigidity in the loop structure (Thr-209 to Cys-214) and the β-sheet structure (Val-217 to Leu-219). Considering the RMSF result analysis, it is apparent that the newly formed hydrogen bond between R216 (Arg-216) and S218 (Ser-218) improved the stability of both the aforementioned loop structure and the β-sheet structure. Pang *et al*. replaced Lys (K) 419 in a type II pullulanase (PulA) adjacent to its (β/α) 8-barrel catalytic domain with Arg (R), introduced a hydrogen bond between Arg-419 and aspartic acid 416 and eliminated the adverse effect of the unstable long side chain of Lys-419 on its catalytic domain, yielding the K419R mutant with improved activity and thermostability [21].

**Fig. 5:**
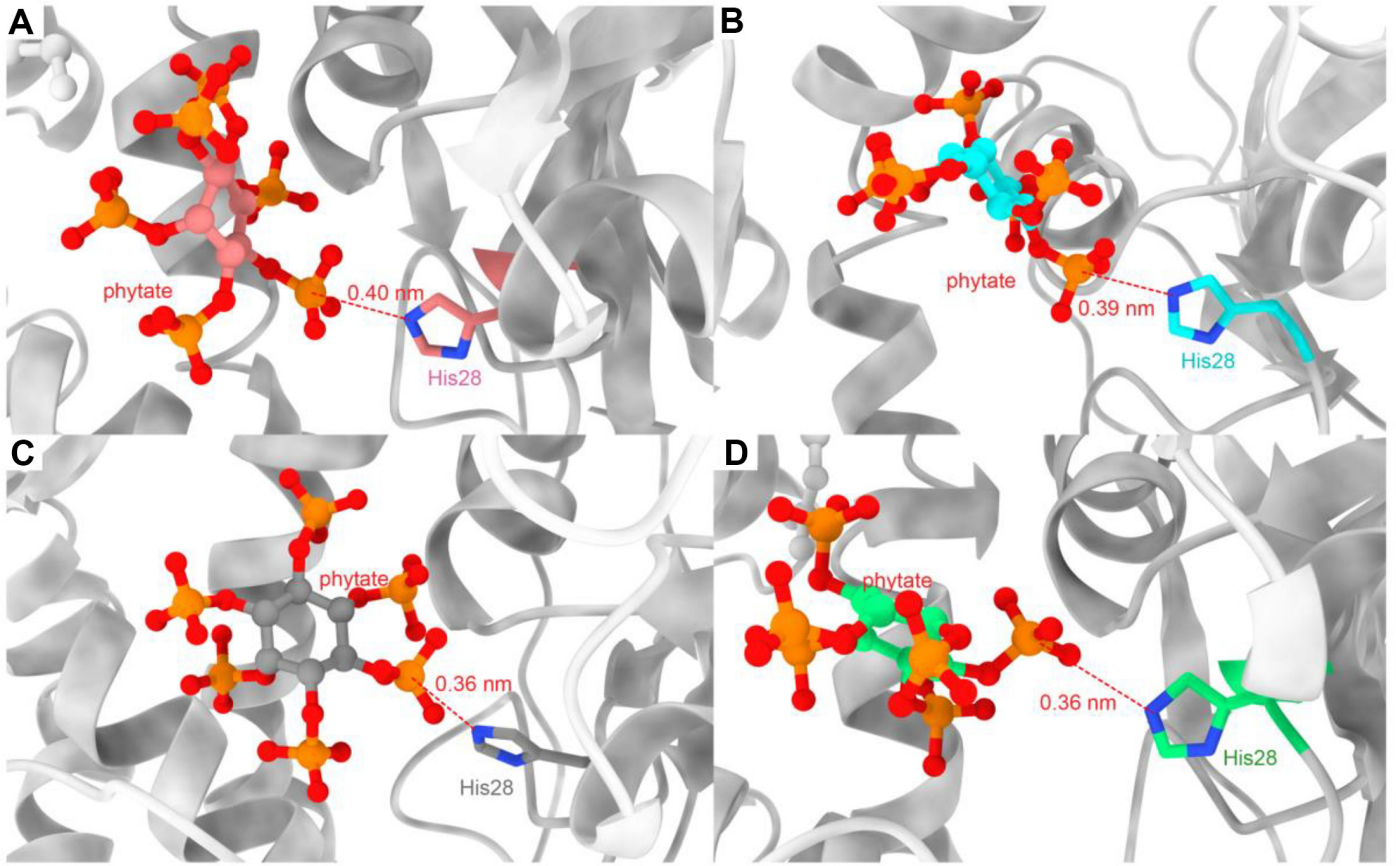
The nucleophilic attack distance between His28 in recombinant phytase and phytate substrate. (**A**) YiAPPA. (**B**) K189R. (**C**) K216R. (**D**) K189R/K216R. The red dotted line indicates atomic distance.

To further explore the molecular mechanism underlying the increased enzymatic activity of the mutant, the MMPBSA method was employed to calculate and decompose the total binding free energy of recombinant phytase and substrate phytate. Solvation energy refers to the sum of polar and non-polar solvation energy. The solvation energy values of YiAPPA and its mutants K189R, K216R, and K189R/K216R were 2055.79, 2072.31, 2098.97, and 2296.54 kcal/mol, respectively (Table 3). Theoretically, higher solvation energy is not conducive to substrate and enzyme binding [38]. The solvation energy of K216R and K189R/K216R, which displayed significantly improved enzymatic activity, was greater than that of YiAPPA. However, the absolute values of van der Waals energy and electrostatic energy for the two mutants were higher than those of YiAPPA, which was sufficient to offset the adverse effects of solvation during enzyme-substrate binding. The total binding energy of the K189R mutant was -67.29 kcal/mol, similar to that of YiAPPA. The total binding energy of the K216R mutant was -92.07 kcal/mol, which decreased by 43.41% compared to that of YiAPPA. The total binding energy of the K189R/K216R mutant was -97.07 kcal/mol, which was reduced by 51.20% relative to YiAPPA. These results show that the mutants K216R and K189R/K216R have increased stability and affinity after substrate binding, which is consistent with the changing trend of *K_m_* values. Additionally, in this study, we structurally analyzed the nucleophilic attack distance between the catalytic amino acid residue His28 and the C6 phosphate group of the phytate substrate; the results are shown in Fig. 5. In the K189R mutant, the nucleophilic attack distance between His28 and the C6 of the phytate substrate was 0.39 nm (Fig. 5B), basically consistent with that of YiAPAP (Fig. 5A). In the K216R and K189R/K216R mutants, this distance was shortened to 0.36 nm and 0.36 nm, respectively (Fig. 5C and 5D). The shorter nucleophilic attack distance between His28 and the C6 of the phytate substrate is conducive to enhancing substrate affinity. The above results demonstrated that the decrease in the total binding energy of the enzyme and substrate, along with the shorter nucleophilic attack distance between the catalytic amino acid His28 and the C6 phosphate group of the phytate substrate, significantly improved the substrate affinity of the mutant. This was the main reason for the enhanced enzymatic activity of the mutant.

### Application of the Recombinant Phytase as a Biocatalyst in Dephytinization of Food Ingredients

The phytate in food ingredients (wheat, corn, and soybean flours) was hydrolyzed by the recombinant phytase at 55°C. After hydrolysis, the content of inorganic phosphorus in the sample and the phytate degradation rates were measured and shown in Table 4. After the treatment of recombinant phytase YiAPPA and its K189R/K216R mutant, the content of inorganic phosphorus in the food ingredients and its phytate degradation rate were enhanced. In addition, compared with that of YiAPPA, the phytate hydrolysis efficiency of K189R/K216R (based on the inorganic phosphorus content in samples) against the food ingredients was increased by 2.36 (wheat flour), 2.28 (corn flour), and 1.73 times (soybean flour), respectively. After treatment with K189R/K216R at 55°C for 2 h, the inorganic phosphorus content in the wheat, corn, and soybean flour samples was 20.14 (wheat flour), 13.04 (corn flour), and 27.54 mg/g (soybean flour) with phytate degradation rates of 79.12%, 57.59%, and 81.52%, respectively. The above results reveal that the K189R/K216R mutant could more effectively reduce phytate in food ingredients and release inorganic phosphorus than YiAPPA, eliminating the anti-nutritional effect of phytate and improving the absorption and utilization of phosphorus in food.

This study successfully created the phytase mutant K189R/K216R with improved thermostability through protein surficial residue mutation. The *t*_1/2_ of K189R/K216R at 55°C was 60.45 min, which was increased by 1.01 times, and its *t*_1/2_ at 80°C was 23.35 min, which was 0.58 times longer than that of YiAPPA. The *T*_50_^30^ of K189R/K216R was 62.44°C, which was 7.32°Chigher than that of YiAPPA, and its *T*_m_ was 53.18°C, which was 4.82°C higher than that of YiAPPA. Furthermore, the specific activity of K189R/K216R was 4469.13 U/mg, which was 0.13 times higher than that of YiAPPA. The analysis of molecular structure modeling and molecular dynamics simulations revealed that the enhanced thermostability of the K189R/K216R mutant, compared to that of YiAPPA, is due to the increased number of hydrogen bonds between amino acid residues and enhanced conformational rigidity of specific enzyme molecular structures. Of note, the improved enzymatic activity of the K189R/K216R mutant was mainly attributed to the decreased enzyme-substrate binding energy and the shorter distance of nucleophilic attack between the catalytic residue His28 and the phytate substrate. This study indicated that protein surficial residue mutation effectively improved the thermostability of the phytase YiAPPA. Additionally, the K189R/K216R mutant exhibited a 1.73–2.36-fold increase in the hydrolysis efficiency of phytate in food ingredients compared to YiAPPA. This study provides essential evidence for the molecular modification of phytase and other enzymes, laying the foundation for applying YiAPPA in food processing.

## Supplemental Materials

Supplementary data for this paper are available on-line only at http://jmb.or.kr.



## Figures and Tables

**Table 1:** Surficial Lys and Gly residues of YiAPPA predicted using GetArea.

Amino acid	Surface exposure ratio (%)	Amino acid	Surface exposure ratio (%)
Gly16	100.0	Lys171	97.2
Lys35	92.9	Lys189	73.0
Gly81	72.7	Gly195	85.7
Gly86	67.3	Lys196	69.8
Gly117	100.0	Lys207	77.5
Gly119	77.3	Lys212	100.0
Lys121	79.7	Lys216	74.6
Lys129	96.4	Lys378	97.6
Lys130	68.1	Lys382	86.9
Lys144	70.8	Gly395	71.2
Lys152	72.2	Gly400	75.7
Gly159	81.2	Lys412	74.0
Lys167	99.4		

**Table 2:** Specific activity (37°C), kinetic parameters (37°C), thermodynamic parameters, and protease resistance of the recombinant phytase.

Recombinant phytase	Specific activity (U/mg)	*K_m_* (μM)	*kcat* (s^-1^)	*kcat* / *K_m_* (μM^-1^ s^-1^)	55°C *t*_1/2_ (min)	80°C *t*_1/2_ (min)	*T*_50_^30^ (°C)	*T_m_* (°C)	Pepsin resistance (%)	Trypsin resistance (%)
YiAPPA	3960.81 ± 105.34	126.24 ± 14.18	10375.27 ± 1256.73	82.19	30.09 ± 0.19	14.81 ± 0.37	55.12 ± 0.87	48.36 ± 0.53	84.17 ± 3.20	82.28 ± 4.18
K189R/K216R	4469.13 ± 154.19	111.59 ± 11.03	10363.29 ± 987.56	92.87	60.45 ± 0.63	23.35 ± 0.57	62.44 ± 0.76	53.18 ± 0.62	85.43 ± 4.09	83.97 ± 3.57
K189R	3979.56 ± 121.00	125.30 ± 13.09	10301.32 ± 1107.83	82.21	47.34 ± 0.38	17.86 ± 0.51	57.32 ± 0.64	49.93 ± 0.32	84.36 ± 2.98	83.01 ± 5.03
K216R	4352.96 ± 121.15	115.01 ± 9.86	10381.09 ± 1091.32	90.26	50.09 ± 0.52	18.90 ± 0.43	59.85 ± 0.73	50.79 ± 0.57	84.91 ± 3.25	83.30 ± 4.18

**Table 3:** Binding energy and various energy contributions of recombinant phytase and substrate phytate (kcal/mol).

Recombinant phytase	ΔG_vdW_	ΔG_ele_	ΔG_polar_	ΔG_non-polar_	ΔG_bind_
WT	-18.35 ± 2.70	-2101.65 ± 13.67	2059.44 ± 9.03	-3.65 ± 0.04	-64.20 ± 16.60
K189R	-21.96 ± 2.81	-2117.64 ± 7.96	2075.72 ± 2.09	-3.41 ± 0.08	-67.29 ± 8.69
K216R	-18.59 ± 2.53	-2172.44 ± 8.28	2103.10 ± 2.77	-4.13 ± 0.03	-92.07 ± 9.09
K189R/K216R	-19.51 ± 1.93	-2374.11 ± 8.52	2300.54 ± 15.18	-4.00 ± 0.02	-97.07 ± 17.51

**Table 4:** Hydrolysis of phytates in food ingredients by the recombinant phytase.

Food Ingredient	Recombinant Phytase	Inorganic phosphorus (mg/g)			Phytate degradation rate (%)		
		0.5 h	1 h	2 h	0.5 h	1 h	2 h
Wheat flour	YiAPPA	5.09 ± 0.39	7.03 ± 0.28	8.52 ± 0.67	19.99	27.62	33.47
	K189R/K216R	6.65 ± 0.53	15.05 ± 0.56	20.14 ± 0.98	26.13	59.13	79.12
Corn flour	YiAPPA	3.49 ± 0.36	4.89 ± 0.42	5.72 ± 0.37	15.41	21.60	25.26
	K189R/K216R	4.58 ± 0.39	9.92 ± 0.51	13.04 ± 0.49	20.23	43.81	57.59
Soybean flour	YiAPPA	8.94 ± 0.38	14.08 ± 0.51	15.95 ± 0.63	26.46	41.68	47.21
	K189R/K216R	10.08 ± 0.59	21.31 ± 0.74	27.54 ± 0.66	29.84	63.08	81.52
