# Supplementary material for: Engineering of the Phytase YiAPPA to Improve Thermostability and Activity and Its Application Potential in Dephytinization of Food Ingredients
Source: J Microbiol Biotechnol. 2024 Jun 30;34(8):1660–70. doi: 10.4014/jmb.2403.03031 (PMC11380507; doi:10.4014/jmb.2403.03031)
Supplement: Supplementary file 1 [file jmb-34-8-1660-supple.pdf]

J. Microbiol. Biotechnol. 2024. 34(8): 1660–1670
https://doi.org/10.4014/jmb.2403.03031

Supplementary Table

Engineering of the Phytase YiAPPA to Improve Thermostability and Activity and Its Application Potential in Dephytinization of Food Ingredients

Jing Zeng*, Jianjun Guo, and Lin Yuan

Institute of Microbiology, Jiangxi Academy of Sciences, Nanchang 330096, Jiangxi Province, P.R. China

Table S1. Primers used for recombinant plasmid construction.

Primer ID	Sequence
G16A-F	5′-GCCATACAACCCACGGCCTATACATTGGAGCG-3′
G16A-R	5′-CGCTCCAATGTATAGGCCGTGGGTTGTATGGC-3′
K35R-F	5′-GTTCGCTCGCCAACCAGACAAACACAGTTAATG-3′
K35R-R	5′-CATTAACTGTGTTTGTCTGGTTGGCGAGCGAAC-3′
G81A-F	5′-CTTCCGTAGCCAAGCGTTACTCGCAGCAGGGTG-3′
G81A-R	5′-CACCCTGCTGCGAGTAACGCTTGGCTACGGAAG-3′
G86A-F	5′-GTTACTCGCAGCAGCGTGTGGACTGAAAGTAC-3′
G86A-R	5′-GTACTTTCAGTCCACACGCTGCTGCGAGTAAC-3′
G117A-F	5′-GGAATAGCACCGGCGTGTGGACTGAAAGTAC-3′
G117A-R	5′-GTACTTTCAGTCCACACGCCGGTGCTATTCC-3′
G119A-F	5′-GCACCGGGGTGTGCACTGAAAGTACATTATC-3′
G119A-R	5′-GATAATGTACTTTCAGTGCACACCCCGGTGC-3′
K121R-F	5′-CGGGGTGTGGACTGAGAGTACATTATCAGGCTG-3′
K121R-R	5′-CAGCCTGATAATGTACTCTCAGTCCACACCCCG-3′
K129R-F	5′-CAGGCTGATTTGAGAAAAGTGGATCCGCTG-3′
K129R-R	5′-CAGCGGATCCACTTTTCTCAAATCAGCCTG-3′
K130R-F	5′-GGCTGATTTGAAAAGAGTGGATCCGCTGTTTC-3′
K130R-R	5′-GAAACAGCGGATCCAVTCTTTTCAAATCAGCC-3′
K144R-F	5′-CGACGCGGGGGTGTGTAGGTTAGATTCGACAC-3′
K144R-R	5′-GTGTCGAATCTAACCTACACACCCCCGCGTCG-3′
K152R-F	5′-CGACACAAACCCATAGGGCTGTTGAGGAGCGAC-3′
K152R-R	5′-GTCGCTCCTCAACAGCCCTATGGGTTTGTGTCG-3′
G159A-F	5′-GAGGAGCGACTAGCTGGGCCATTAAGTG-3′
G159A-R	5′-CACTTAATGGCCCAGCTAGTCGCTCCTC-3′
K167R-F	5′-GTGAACTGAGCAGACGCTATGCTAAG-3′
K167F-R	5′-CTTAGCATAGCGTCTGCTCAGTTCAC-3′
K171R-F	5′-CAAACGCTATGCTAGGCCCTTTGCCCAGATG-3′
K171R-R	5′-CATCTGGGCAAAGGGCCTAGCATAGCGTTTG-3′

K189R-F	5′-CTCCTTACTGTAGATCACTGCAACAGCAAG-3′
K189R-R	5′-CTTGCTGTTGCAGTGATCTACAGTAAGGAG-3′
G195A-F	5′-CACTGCAACAGCAAGCGAAAACCTGTGATTTTG-3′
G195A-R	5′-CAAAATCACAGGTTTTCGCTTGCTGTTGCAGTG-3′
K196R-F	5′-CTGCAACAGCAAGGGAGAACCTGTGATTTTG-3′
K196R-R	5′-CAAAATCACAGGTTCTCCCTTGCTGTTGCAG-3′
K207R-F	5′-GTTGCAGCGAATAGGATCACGGTGAACAAG-3′
K207R-R	5′-CTTGTTCACCGTGATCCTATTCGCTGCAAC-3′
K212R-F	5′-GATCACGGTGAACAGGCCGGGGACAAAAG-3′
K212R-R	5′-CTTTTGTCCCCGGCCTGTTCACCGTGATC-3′
K216R-F	5′-CAAGCCGGGGACAAGAGTCTCGCTCAGC-3′
K216R-R	5′-GCTGAGCGAGACTCTTGTCCCCGGCTTG-3′
K378R-F	5′-GCGAAATGCTGAGAGACTAGACCTGAAAAAC-3′
K378R-R	5′-GTTTTTCAGGTCTAGTCTCTCAGCATTTCGC-3′
K382R-F	5′-GAAACTAGACCTGAGAAACAATCCGGCTGGTAG-3′
K382R-R	5′-CTACCAGCCGGATTGTTTCTCAGGTCTAGTTTC-3′
G395A-F	5′-CTGTTGCAATAGACGCTTGTGAAAATAGTGGTG-3′
G395A-R	5′-CACCACTATTTTCACAAGCGTCTATTGCAACAG-3′
G400A-F	5′-GTGAAAATAGTGCTGATGACAAACTTTGTC-3′
G400A-R	5′-GACAAAGTTTGTCATCAGCACTATTTTCAC-3′
K412R-F	5′-GATACCTTCCAAAGGAAAGTAGCTCAGGCG-3′
K412R-R	5′-CGCCTGAGCTACTTTCCTTTGGAAGGTATC-3′

Note: The bases marked in a box are mutated bases.
